# Quality-by-Design Is a Tool for Quality Assurance in the Assessment of Enantioseparation of a Model Active Pharmaceutical Ingredient

**DOI:** 10.3390/ph13110364

**Published:** 2020-11-04

**Authors:** Dina Aboushady, Maria Kristina Parr, Rasha S. Hanafi

**Affiliations:** 1 Department of Pharmaceutical Chemistry, Faculty of Pharmacy and Biotechnology, German University in Cairo, Cairo 11835, Egypt; dina.abou-shady@guc.edu.eg (D.A.); rasha.hanafi@guc.edu.eg (R.S.H.); 2 Institute of Pharmacy, Freie Universität Berlin, Königin-Luise-Str. 2 + 4, 14195 Berlin, Germany

**Keywords:** quality-by-design, quality assurance, design-of-experiments, chiral separation, chiral mobile phase modifier, terbutaline

## Abstract

The design of experiments (DoE) is one of the quality-by-design tools valued in analytical method development, not only for cost reduction and time effectiveness, but also for enabling analytical method control and understanding via a systematic workflow, leading to analytical methods with built-in quality. This work aimed at using DoE to enhance method understanding for a developed UHPLC enantioseparation of terbutaline (TER), a model chiral drug, and to define quality assurance parameters associated with using chiral mobile phase additives (CMPA). Within a response surface methodology workflow, the effect of different factors on both chiral resolution and retention was screened and optimized using Plackett-Burman and central composite designs, respectively, followed by multivariate mathematical modeling. This study was able to delimit method robustness and elucidate enantiorecognition mechanisms involved in interactions of TER with the chiral modifiers. Among many CMPAs, successful TER enantioresolution was achieved using hydroxypropyl β-cyclodextrin (HP-β-CD) added to the mobile phase as 5.4 mM HP-β-CD in 52.25 mM ammonium acetate. Yet, limited method robustness was observed upon switching between the different tested CMPA, concluding that quality can only be assured with specific minimal pre-run conditioning time with the CMPA, namely 16-column volume (60 min at 0.1 mL/min). For enantiorecognition understanding, computational molecular modeling revealed hydrogen bonding as the main binding interaction, in addition to dipole-dipole inside the CD cavity for the *R* enantiomer, while the *S* enantiomer was less interactive.

## 1. Introduction

**Figure 1:**
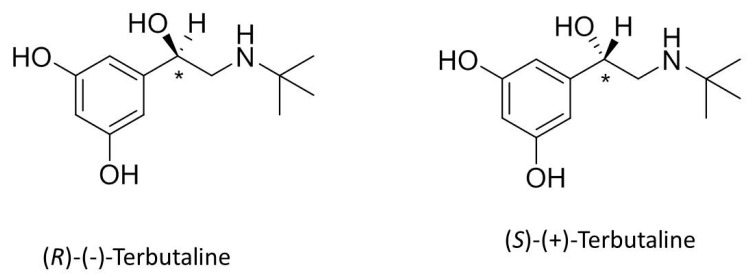
Chemical structures of *R*- and *S*-enantiomers of terbutaline (TER).

The pharmaceutical industry is obliged to adopt quality-by-design (QbD) strategies as per the International Council for Harmonisation of Technical Requirements for Pharmaceuticals for Human Use (ICH) guidelines Q8, Q9, and Q10, in order to ensure robust manufacturing processes and enhanced product quality. The concepts of QbD have been extrapolated to the development and verification of analytical methods in what is known as analytical quality-by-design (AQbD) [1,2,3,4]. Design of experiments (DoE) is one of the tools of AQbD where multivariate statistical analysis is used for the optimization of analytical method conditions [5,6]. DoE mathematically determines main factor effects, as well as their interactions, on chromatographic separation and retention responses with a predefined number of experiments. The mathematical model and the graphical response surface allow superior, global, and accurate understanding of the simultaneous effects of variable factors on the measured response, minimizes costs and time spent during method development, and allows visualization of the design space or response surface [7,8,9]. DoE is applied for the optimization of a wide range of chromatographic methods, including chiral separations [9,10,11,12,13,14]. Instead of using expensive, widely available chiral stationary phases (CSP) [14,15,16,17,18], limited budget laboratories of small industrial pharmaceutical enterprises use chiral mobile phase additives (CMPA) to achieve enantioresolution on commonly available achiral stationary phases in various modes (normal phase and reversed phase) [19,20,21,22,23]. Analogous approaches are commonly used in capillary electrophoresis [24,25,26,27]. Terbutaline (TER) (Figure 1) is a selective β2-agonist, therapeutically used as a fast-acting bronchodilator and tocolytic agent. It is marketed by many pharmaceutical companies as a racemic mixture (*rac*TER) i.e., an equal ratio of the (*R*) eutomer and (*S*) distomer [28,29]. In 1992, the FDA highlighted that the determination of the different enantiomers is necessary and cannot be ignored since the enantiomers of a biologically active chiral compound often behave differently in pharmacokinetic and pharmacological activity. Guidelines request the consideration of each enantiomer of pharmaceuticals as single active compounds [30]. Hence, TER enantiomers were chiraly resolved—among many methodologies—using native and substituted beta cyclodextrins as CMPA by capillary electrophoresis (CE) and HPLC [25,31,32,33,34]. Despite the availability of numerous reports regarding TER enantioseparation in the literature, no understanding of the mechanistics of chiral recognition has ever been reported [15,35,36], likely because all reports use a one-factor-at-a-time (OFAT) approach during optimization, leaving factors’ interactions unexamined. This old OFAT method development strategy risks failure, especially if the analyst aims at method transfer, where risk-based approaches for process developments are indeed recommended by the FDA [37,38]. Thus, the aim of this study is to shed the light on the enantiorecognition mechanism of TER using CMPA via a QbD approach using DoE, evaluate method robustness as a part of quality assurance for chiral analysis of TER by CMPA in a pharmaceutical industry context, and to delineate the response surface for optimum chromatographic conditions, achieving the highest resolution and least retention.

## 2. Results

### 2.1. Screening Design

**Figure 2:**
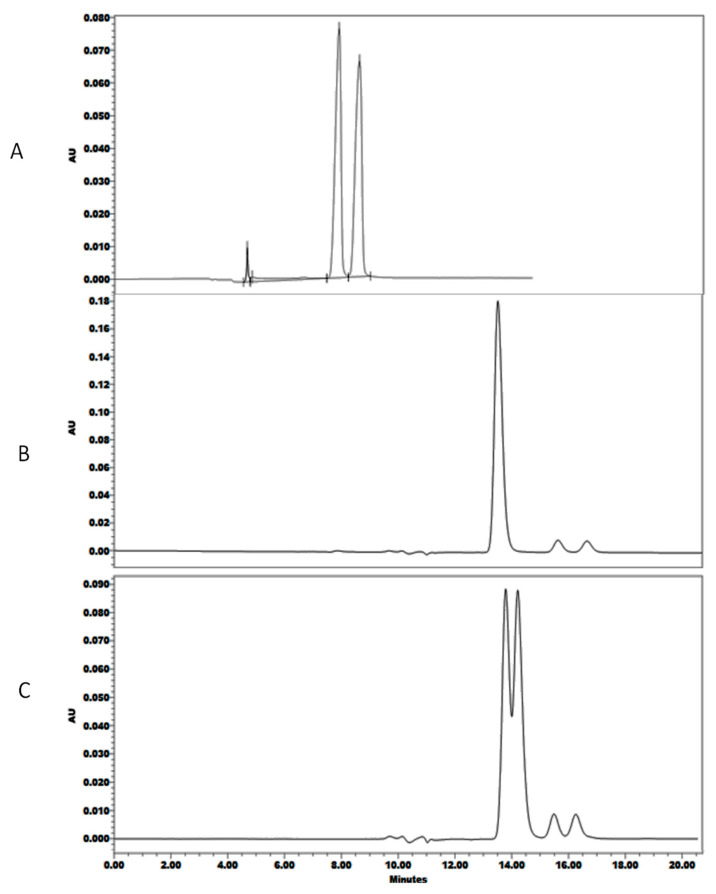
UHPLC chromatograms of racemic Terbutaline (*rac*TER) standard with mobile phase conditions: (**A**) 2.3 g/L sulfobutylether β-cyclodextrin (SBE-β-CD) in 0.05% formic acid: ACN, isocratic elution (93:7) at 0.3 mL/min achieving tR_Last_ at 8.5 min and α = 1.09; (**B**) 8 mmol/L hydroxypropyl β-cyclodextrin (HP-β-CD) in 0.05 M ammonium acetate: MeOH adjusted to pH 6, isocratic elution (95:5) at flow rate: 0.1 mL/min achieving tR_Last_ 13.5 min and α = 1; (**C**) 8 mmol/L HP-β-CD in 0.05 M ammonium acetate: MeOH adjusted at pH = 4, isocratic elution (95:5) at flow rate: 0.1 mL/min achieving tR_Last_ 14.2 min and α = 1.03. The two small peaks were artifacts appearing in all chromatograms.

Among the screened CMPA, two were promising: hydroxypropyl β-cyclodextrin (HP-β-CD) and sulfobutylether β-cyclodextrin (SBE-β-CD) with degree of substitution (ds) of about6, where tR_Last_ was 8.5 min and α was 1.09 upon using SBE-β-CD (Figure 2A), while HP-β-CD resulted only in partial enantioseparation of TER when the pH was adjusted to 4 (Figure 2C) and no separation at pH 6 (Figure 2B). Experimental run conditions and corresponding results of retention and separation are summarized in Table 1. Main effects plots revealed that among the six screened factors, three were most influential on targeted responses, namely type and concentration of the CMPA, in addition to the concentration of buffer (Supplementary Materials Figure S1). Moreover, Pareto charts showed the cumulative effect of these three factors on responses (Figure S2), supporting their incorporation in the following optimization design.

### 2.2. Optimization Design

A circumscribed central composite design (CCD) was selected to demarcate the design space of TER chiral separation via 26 chromatographic runs of different levels for each of the three studied factors (Table 2).

### 2.3. Multiple Linear Regression Models

**Figure 3:**
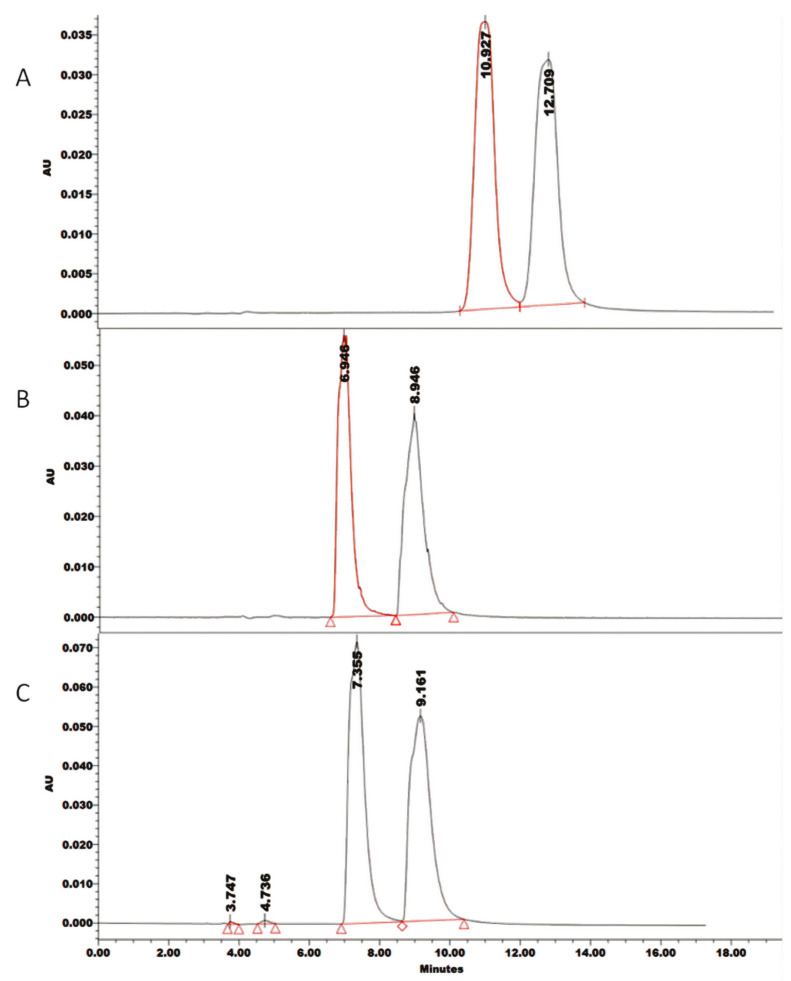
Chromatograms for three randomly selected experimental conditions within the optimum robust surface of contour plots to validate the prediction power of regression models in Equations (3) and (4); (**A**) 5 mM SBE-β-CD in 5 mM ammonium acetate: ACN (97:3) pH = 3.5 at 0.3 mL/min achieving tR_Last_ 12.7 min and α = 1.17 (predicted α = 1.18); (**B**) 5.4 mM HP-β-CD in 5 mM ammonium acetate: ACN (97:3) at pH = 3.5 at 0.3 mL/min achieving tR_Last_ 8.9 min and α = 1. 28 (predicted α = 1.30); (**C**) 3 mM HP-β-CD in 50 mM ammonium acetate: ACN (97:3) at pH = 3.5 at 0.3 mL/min achieving tR_Last_ 9.1 min and α = 1.24 (predicted α = 1.25).

The regression model was computed for tR_Last_ and α, where optimal Box-Cox transformations of the response were ln tR_Last_ and α (λ − 1)/((λ × g^(λ − 1))”, where λ = 3 and g = 1.16737 as the geometric mean of α (Equations (1)–(4)). Model fitting was evaluated using values of correlation coefficients (R^2^), while the absence of an insignificant term was tested by ANOVA (*p*-values < 0.05). The regression models obtained were validated experimentally and results for both types of CMPA successfully met the predicted values (Figure 3).

Equations (1) and (2) imply that, for both types of CMPA, a shorter retention time is only dependent on buffer concentration. The enhancement of elution by the buffer is attributed to the ionic interaction of NH_4_Ac with TER in the mobile phase as a result of the ionization of the TER tertiary amine group and its interaction with the acetate group at the studied pH. It also leads to improved peak shape due to the blockage of the free silanol groups of the stationary phase by the ammonium group in the buffer.

For HP-β-CD:

(1) $$\ln\left({\mathrm{tR}}_{\mathrm{Last}}\right)=\;1.657\;+\;0.0603\;\mathrm{Buffer}\;\mathrm{conc}\;-\;0.000979{\;\mathrm{Buffer}\;\mathrm{conc}}^{2}$$

For SBE-β-CD:

(2) $$\ln\left(\mathrm{tRLast}\;\right)=\;2.686\;+\;0.0603\;\mathrm{Buffer}\;\mathrm{conc}\;-\;0.000979{\;\mathrm{Buffer}\;\mathrm{conc}}^{2}$$

In Equations (3) and (4), selectivity is influenced by CMPA concentration and buffer concentration for both types of CMPA. Factors were shown to affect selectivity in their first and second order.

For HP-β-CD:

(3) $$\begin{array}{ll} \frac{\alpha^{\lambda -1}}{\lambda \times g^{\left(\lambda -1\right)}} & =\;0.1816+\;0.0569\;\mathrm{CMPA}\;\mathrm{conc}\;-0.00935\;\mathrm{Buffer}\;\mathrm{conc}\\ & -\;0.00524{\;\mathrm{CMPA}\;\mathrm{conc}}^{2}\;+\;0.000182{\;\mathrm{Buffer}\;\mathrm{conc}\;}^{2} \end{array}$$

(λ = 3, g = 1.16737 is the geometric mean of alpha α)

For SBE-β-CD:

(4) $$\begin{array}{ll} \frac{{\;\alpha }^{\lambda -1}}{\lambda \times g^{\left(\lambda -1\right)}} & =\;0.0041+\;0.0569\;\mathrm{CMPA}\;\mathrm{conc}\;+0.00186\;\mathrm{Buffer}\;\mathrm{conc}\\ & -0.00524{\;\mathrm{CMPA}\;\mathrm{conc}}^{2}-0.000047{\;\mathrm{Buffer}\;\mathrm{conc}\;}^{2} \end{array}$$

(λ = 3, g = 1.16737 is the geometric mean of alpha α)

**Figure 4:**
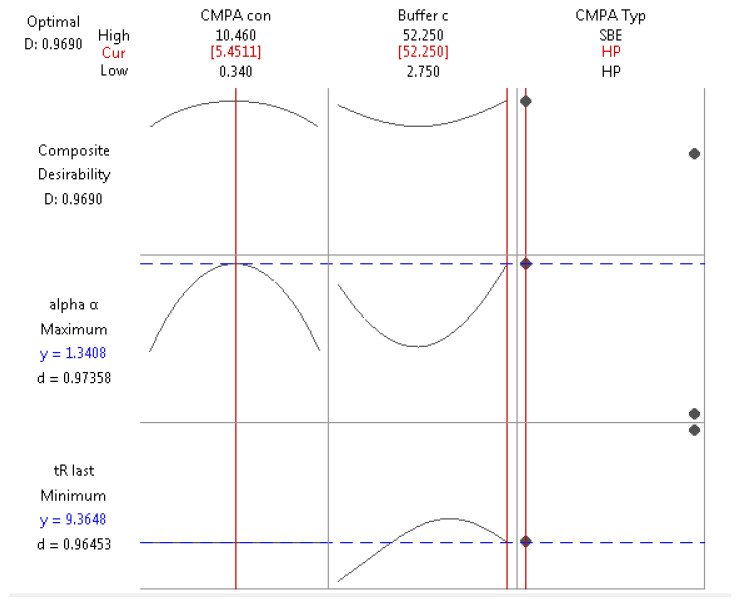
Optimization plots of factors vs. responses, where each column describes the effect of individual factors of the regression Equations (1)–(4) on the measured responses α and tR_Last_. Optimum conditions that simultaneously achieve maximum α and minimum tR_Last_ are 5.4511 mM HP-β-CD and 52.250 mM buffer in a global desirability (D) of 96.90%. Lines and values in red show the factor points included in the optimized response conditions, Horizontal blue lines shows the desirability function values achieving maximum α and minimum tR_Last_.

To obtain a compromise in conditions leading to maximum α and minimum tR_Last_, the global desirability function (D) was used (Figure 4). HP-β-CD at a concentration of 5.45 mM in aqueous 52.25 mM NH_4_Ac successfully resulted in an experimental least retention time of 9.6 min within a 95% confidence interval (7.52–13.95 min) and highest experimental α of 1.22 at a 95% predicted interval (1.212–1.449) (Figure 4).

## 3. Discussion

In the screening design, the steepness of the lines in the main effect plots (Figure S1) show that the use of an acidic mobile phase and low %B had a positive effect on enantioresolution, indicating that the lower the pH and %B, the higher the enantioseparation. Changing the flow rate between the studied levels (0.05 to 0.3 mL/min) did not significantly affect the separation, but definitely shortened retention time. The low ionic strength of the buffer had the benefits of improving enantioseparation, fastening elution, and preventing precipitation of buffer salt when the aqueous and organic phases meet in the UHPLC mixing chamber. Doubling the rate of the change in %B in the linear gradient caused a noticeably shorter retention time due to the increased eluent strength of the mobile phase, according to the linear solvent strength theory, indicating that hydrophobic–hydrophobic interactions are governing analyte (stationary phase) mobile phase interactions. However, high %B decreased the solubility of CMPA and worsened enantioseparation, likely due to competition of the organic modifier with the chiral binding site at the β-CD cavity as reported by Gratz et al [25]. Finally, the mobile phase was set at pH = 3.5 and %B change at 0.02 as fixed conditions for all upcoming runs of the optimization design.

The linear solvent strength theory governing reversed phase (RP) separations is known to be a logarithmic relationship where the retention factor K to the base 2 is related to surface tension of the aqueous mobile phase modifier (ln K = A + BD+ Cɣ+ D(Ke − 1)V2/3ɣ + E + ln RT/PoV, where A, B, C and D are constants). Our regression model for retention (Equations (1) and (2)) has the same logarithmic relationship to the base 2, which infers that hydrophobic interactions predominate in the three-point interaction of enantiomers, leading to chiral separation even in the presence of the CMPA in the mobile phase. Moreover, faster elution was possible with large %B change (0.04%/min), supporting the fact that reversed phase mode is followed in the presence of CMPA. On the other hand, selectivity (α) is dependent on buffer and CMPA concentrations in first and second orders (Equations (3) and (4)). Hence, careful selection of levels of these two factors is important for quality assurance of the separation, where distancing from the robust optimum values would lead to a dramatic drop in enantioresolution due to the presence of the quadratic terms.

**Figure 5:**
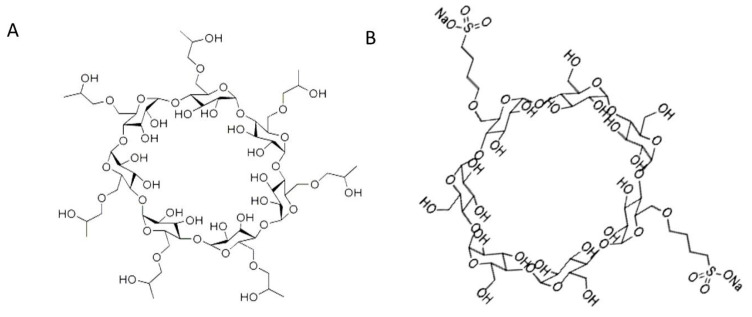
Chemical structure of the CMPA used: (**A**) hydroxypropyl β-cyclodextrin (HP-β-CD); (**B**) sulfobutylether β-cyclodextrin (SBE-β-CD).

**Figure 6:**
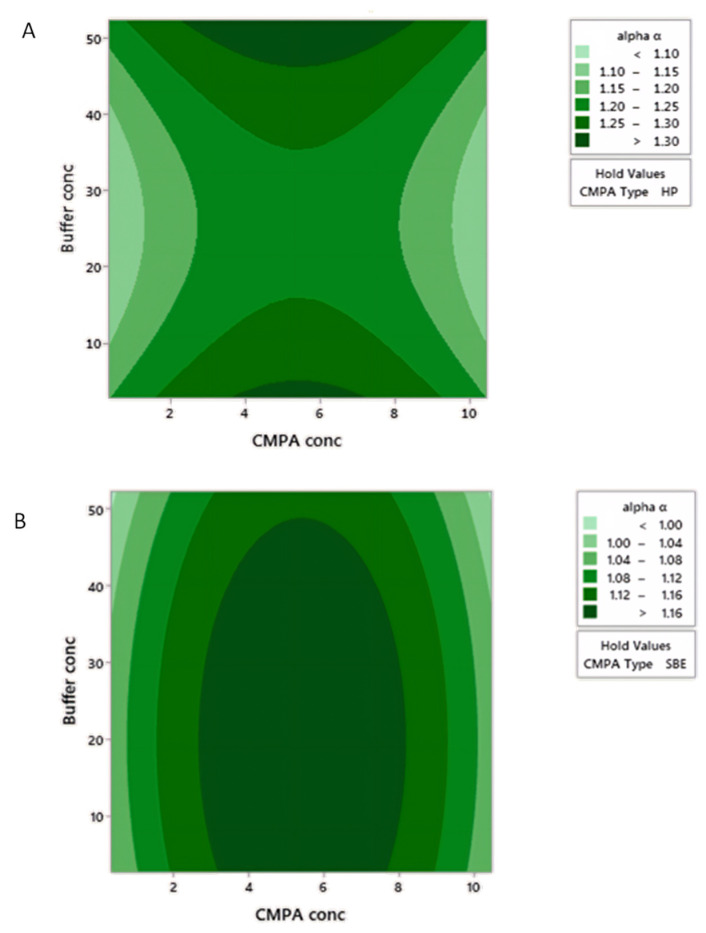
Two-dimensional contour plot for the regression model of separation factor α showing the two variables in the regression equation on the X- and Y-axis of: (**A**) HP-β-CD (**B**) SBE β-CD. Shades of green color indicate different values of α, where darkest is the highest value and vice versa.

HP-β-CD (Figure 5A) displays free rotating substituted hydroxy groups that promote strong and stereospecific interaction via hydrogen bonding with the 3-hydroxy groups of TER, in addition to the inclusion of its aromatic ring into the CD cavity, leading to a hydrophobic interaction. In contrast, enhanced enantioseparation was only possible at moderate CMPA concentrations (4–7 mM) of HP-β-CD (Figure 6A), where higher concentrations showed decreased column effectiveness due to increased column pressure. Despite the fact that the regression model of α with HP-β-CD did not show any interaction terms between buffer concentration and CMPA concentration (Equation (3)), separation was only achieved at either very low or very high concentrations of buffer due to the quadratic order of this factor (Equations (3) and (4)). Moreover, a wider range of CMPA concentration (3–8 mM), concomitantly with the highest buffer concentration (45–50 mM), leads to acceptable separation. This may conclude the involvement of both stationary and mobile phases in which interaction and saturation of the stationary phase with HP-β-CD takes place at low buffer concentration. On the other hand, at high buffer concentration and ionic strength (45–50 mM), the ionized TER predominates in the CMPA rich mobile phase; thus, chiral recognition takes place via both surface stationary phase and mobile phase. This also explains the improved resolution with lower retention time at high buffer concentrations when TER elutes with the HP-β-CD in the mobile phase.

For the sulfonated SBE β-CD (Figure 5B), a different pattern in the relationship between α and both CMPA and NH_4_Ac concentrations was observed. A moderate concentration (3–8 mM) of SBE-β-CD led to a good separation at a wide range of buffer concentration (5–50 mM, Figure 6B). A mixed modal interaction involving ion pairing, hydrogen bonding, and inclusion complex at the hydrophobic cavity are most likely occurring [33]. Separation in a slightly acidic medium (pH = 3.5) guarantees the unionization of some sulfonic groups (pKa = 2), where partitioning with the reversed phase takes place along with the presence of some other ionized sulfonic groups eluting in the mobile phase paired with TER.

As per Equations (3) and (4), separation is weakly affected by buffer concentration (coefficients related to buffer concentration are 6–110 times smaller than coefficients of CMPA concentration) and a wide range of buffer concentration prospers to achieve α > 1.16 (Figure 6B). For similar methods published in literature, no selectivity was reported. On the other hand, resolution was measured instead [33], or it was stated as no separation for TER was achieved on octadecylsilane with the use of HP-βCD in the mobile phase [31]. Other reports using native beta cyclodextrins as CMPA rather than using substituted ones showed slightly lower selectivity values for TER to the values in our study [32]. Our findings contradict the report of Ngim et al. [33], which states that separation was not sensitive to the degree of substitution of CMPA, pH, nor the organic modifier.

In general, HP-β-CD resulted in higher α value than SBE-β-CD on an octadecylsilane column in the presence of an organic modifier in the mobile phase. Thus, HP-β-CD was selected for further response optimization experiments. This contradicts the report by Ameyibor et al. [31], in which TER enantiomers are separated using HP-β-CD in 50 mM NH_4_Ac only on hexylsilane and octylsilane columns with a complete loss of resolution upon the addition of the organic phase when an octadecylsilane column was used.

**Figure 7:**
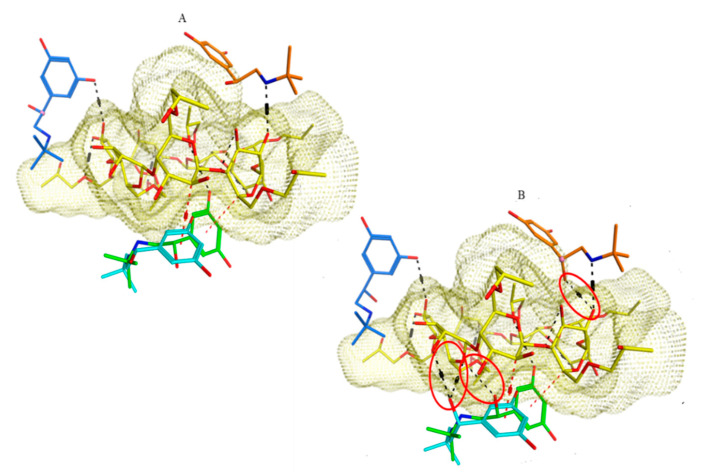
Three-dimensional molecular visualization showing the predicted binding modes upon docking in HP-β-CD (yellow surface). Dotted red lines denote dipole interactions, while dotted black lines denote hydrogen bonding. (**A**) Interactions of four different *S*-TER conformations with the pocket (blue, cyan, green and orange structures), (**B**) docking of four different *R*-TER conformations anchored to the pocket by extra hydrogen bonding at the chiral center (oval red shapes).

Computational docking of different TER conformers into HP-β-CD confirmed the predicted interactions in some favorable docking poses (Figure 7). Interactions inside the CD cavity include hydrogen bonding and dipole interactions between the cavity and the electron cloud of the TER aromatic ring, as well as hydrogen bonding with the externally protruding hydroxypropyl substituent of HP-β-CD. *R*-TER showed additional bonds at the chiral center (Figure 7B) that allow three-point interactions, leading to superior chiral recognition compared to all conformers of *S*-enantiomers.

**Figure 8:**
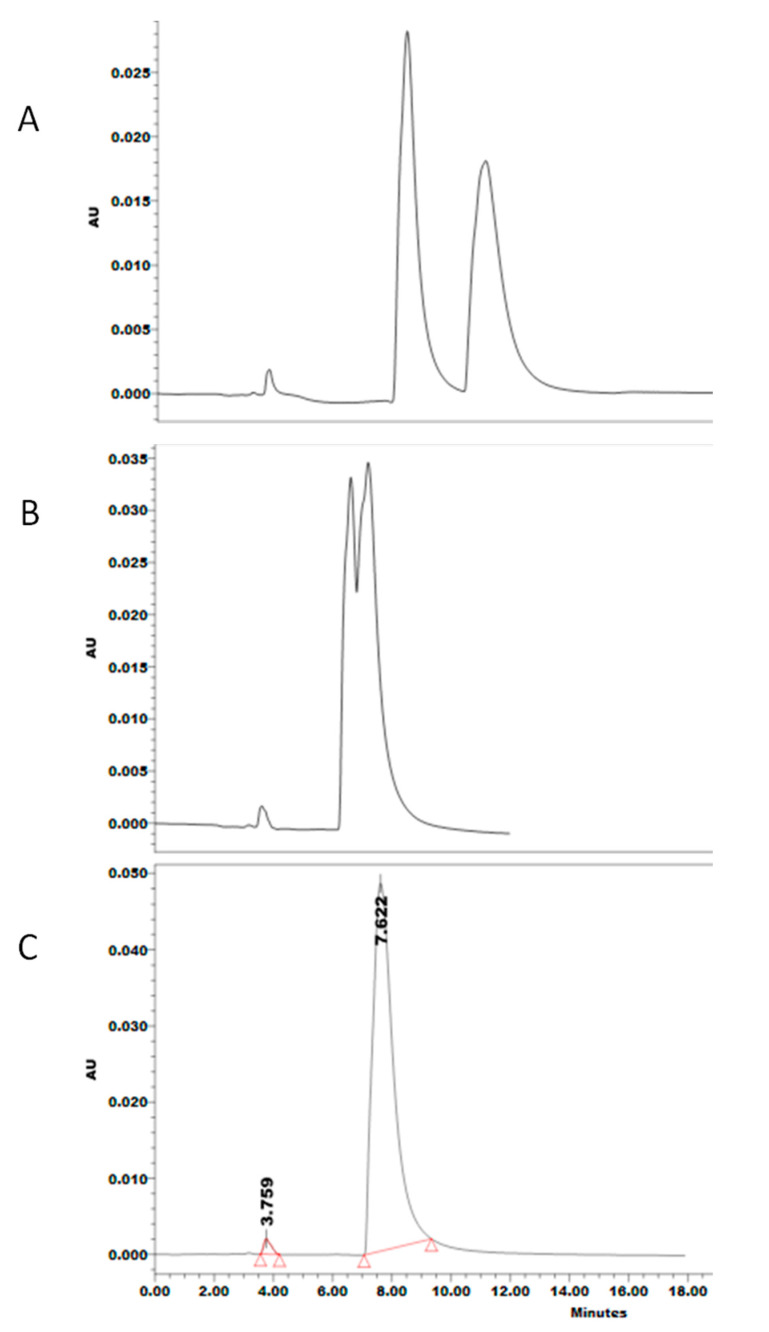
Chromatograms obtained post-washing of the achiral column with the achiral mobile phase (50 mM NH_4_Ac pH 3: ACN, 97:3): (**A**) 5.6×, (**B**) 7.25×, and (**C**) 16× the column volume, showing that the memory effects of former chromatographic conditions persist, and that exact guidelines need to be available for column washing and conditioning for CMPA-based enantioseparation to ensure quality and robustness.

The mandatory randomization of runs in a DoE-based experiment revealed that even after washing the achiral column with an achiral mobile phase (50 mM NH_4_Ac pH 3: ACN, 97:3), between runs involving different CMPA (Table 2), enantioselective interactions of a former run are preserved, where TER are still enantioresolved after several washing cycles (Figure 8). This is likely due to the attachment of the CMPA–TER complex to the stationary phase. Indeed, quality assurance of CMPA-based chiral separations seems to globally suffer from a lack of data about strict protocols of column conditioning and inter-run washes upon switching between different chromatographic conditions, which is proven in our study to severely compromise method robustness. In some experiments, variation in retention times for the same run conditions was observed due to memory effects of former chromatographic conditions. For instance, the retention time of each enantiomer in run 19 (Table 2), expressed as mean ± standard deviation, showed 33.3 ± 1.59 s and 41.0 ± 1.83 s. However, inter-run washing and conditioning achieved a retention time of the enantiomers as mean ± 0.07–0.09 s. This authenticates the ability of the built-in-quality gained by using DoE-based method development to detect a lack of robustness in chiral separations. We identified some factors, including effective column conditioning, appropriate washing time, and suitable mobile phase flow rate for conditioning phases during our screening experiments, while previous reports were not attentive to conditioning time nor to flow rate. The C18 surface was freed from the memory effect after the addition of 16× the normal column wash volume.The C18 surface was freed from the memory effect after using a washing volume equivalent to 16 times normal column volume (Figure 8). For quality assurance, studies using CMPA should include clear protocols for washing and conditioning to preserve reproducibility and robustness. Notably, our findings exclude a potential gradient mode chromatography that is indeed seldom used with CMPA. Even though CSP are more common for enantioseparation, it cannot be ignored that the use of CMPA is cost-effective and provides freedom to the analyst to quickly change between different chiral selectors. Moreover, not all chiral selectors are available as chiral columns. Nevertheless, chiral separations are more challenging upon using CMPA in terms of method transfer and performance since it was concluded that long conditions and washing times are a key factor for quality assurance. Future directions include the application of this chiral approach to members of chiral β_2_ agonists produced by the pharmaceutical industry, not only to evaluate predominant interactions leading to enantioseparation, but also to study quality assurance-related parameters in the analytical method.

## 4. Materials and Methods

### 4.1. Standards and Reagents

*rac*Terbutaline (as sulfate salt) was generously offered by SEDICO Pharmaceuticals (Giza, Egypt). Ammonium acetate (NH_4_Ac) was obtained from Fluka Chemie GmbH (Steinheim, Germany). Native β-cyclodextrin (β-CD) and substituted β-cyclodextrins (charged and uncharged β-CD) were purchased from Cyclolab kft. (Budapest, Hungary). Methanol (MeOH) and acetonitrile (ACN), gradient grades for HPLC, were obtained from Merck KGaA (Darmstadt, Germany). Trifluoroacetic acid (TFA) was used for pH adjustment of the mobile phase (Sigma Aldrich, Steinheim, Germany) and Milli-Q water was used from an ELGA Purelab water purification system (UHQ I, High Wycombe, UK).

### 4.2. Instrumentation

The chiral experiments were performed on an Acquity H-class UHPLC System (Waters, Milford, MA, USA) equipped with a quaternary solvent pump and sample manager, a column compartment, a photodiode array detector set at 280 nm, and a degassing system. Separation was conducted in RP mode using a Hypersil^®^BDS C18 column (100 mm × 4.6 mm × 3 µm) from ThermoFisher Scientific (San Jose, CA, USA). The method involves the use of solvent A containing CMPAs (in varying concentrations) dissolved in ammonium acetate (NH_4_Ac) buffer (with varying buffer pH and concentration), and solvent B which is uniquely composed of acetonitrile. The injection volume was 10 μL and temperature of the autosampler maintained at 25 °C.

### 4.3. Software

UHPLC data acquisition and processing was performed using Empower v.2.0 (Waters, Milford, MA, USA). Statistical analysis and modeling was performed by Minitab^®^19 (Statistical Software, Coventry, UK). Computational chemistry and docking were done using the Molecular Operating Environment (MOE) software package (version 2016, Chemical Computing Group, Montreal, QC, Canada).

### 4.4. Standard Preparation

A TER reference standard solution in methanol (0.30 mg/mL) was used as a test solution during optimization. Pre-analytical sample filtration was carried out using a 0.2 μm syringe filter.

### 4.5. Analytical Target Profile

Before method development, the analytical target profile (ATP) was set to achieve baseline separation of TER enantiomers, as well as to shorten the run time. Consequently, the measured responses were the separation factor (α), to express enantioseparation of TER, and retention time of the latest eluting peak (tR_Last_) as an indication of run time. In addition, it was targeted to interpret the obtained response surfaces to comprehend the enantiorecognition process and to determine parameters influencing the quality assurance of the method.

### 4.6. Plackett-Burman Screening Design for UHPLC Analysis

At an early stage of DoE, a screening design was needed to determine the most influential factors on TER enantioseparation. Six factors were screened, namely CMPA type and concentration, buffer pH and concentration, flow rate, and linear gradient as rate of %B change (Table 3). To have a reasonable number of runs in the screening design, only 2 out of 6 explored CMPA were included based on their significantly different natures and on previously reported promising TER enantioresolution [31,33]. Moreover, the enhanced solubility of the substituted β-CD in the aqueous mobile phase rather than native β-CD supported their further incorporation in experiments in order to study the various binding modes resulting from their chemical difference and accomplishing enantioseparation. Low and high values for each of the 6 chromatographic factors reported in the literature were extended to wider limits to explore responses at what analysts believe is the limit for “the optimal”. The substituted CMPAs selected were charged SBE-β-CD (ds of about6) and SBE-β-CD (ds of about 10), in addition to an uncharged hydroxypropyl derivative (HP-β-CD). HP-β-CD was tested at a concentration of 8 mM in 0.05 M NH_4_Ac:MeOH (95:5), while SBE β-CD with ds of about 6 and ds of about 10 were tested at a concentration of 2.30 g/L in 0.05% FA in water:ACN (93:7). The column was thermostated at 30 °C and TFA was used for pH adjustment in all runs due to its favorable ion pair effect on retention and resolution, as well as its contribution in additional interactions [39,40]. Selection of the most influential factors among the 6 screened factors was aided by Pareto charts and main effects plots.

### 4.7. Multiple Linear Regression Analysis

Multiple linear regression (MLR) was used to determine coefficients by which factors affect the responses, the order of this relationship, and the possible existence of 2- or 3-way interactions between the factors. Box-Cox transformations were attempted to produce a normal distribution of curve fitting residuals, minimize standard deviation, and produce the highest coefficients of determination (adjusted R^2^ and predicted R^2^ values) for the transformed responses α (separation factor) and tR_Last_ (retention time of latest eluting peak). The number of terms in each regression model was restricted to the most significant (*p* value < 0.05) via backward elimination of non-significant terms.

Visualization of the response surface corresponding to the mathematical model is presented as 2D contour and 3D surface plots. To validate the quality of predictability power of the regression model, random experimental points were selected in the robust optimal surface for practical implementation, followed by a comparison of experimental vs. predicted values.

### 4.8. 3D Molecular Modeling

Using the Molecular Operating Environment (MOE) software, a conformational search for TER resulted in 5 conformers that were further docked into the HP-β-CD structure, where atoms in the β-CD cavity were selected and defined as a pocket. Docking was done with different TER conformers as a ligand database using the Amber10: Extended Hückel Theory (EHT) force field and the “triangle matcher” as a placement method (number of return poses set to 200), with “Alpha HB” as scoring. Selected docking poses were further used to evaluate binding modes of different conformers with the host molecule.

## 5. Conclusions

In this work, enantioresolution of TER was optimized via a design of experiment approach on UHPLC-UV, where a simultaneous maximum separation factor (1.22) and least run time (9.6 min) were obtained using HP-β-CD as a chiral mobile phase modifier on a C18 column. The robust experimental conditions of the developed method were delimited by contour plots visualizing areas of optimal responses following multiple regression analysis. For quality assurance and enhanced method robustness, a minimum of 16× the normal column wash volume of the achiral column with the achiral mobile phase is essential to eliminate the memory effects of enantioselectivity produced by CMPA in isocratic mode and achieve retention times expressed as mean ± 0.09. Meanwhile, the need for sufficient conditioning time and molecular docking support the hypothesis that modification of the stationary phase surface via attachment of the CMPA–TER complex to the stationary phase is probable as the mechanism of enantiorecognition. The predominant interactions achieving chiral recognition of TER were hydrogen bonding and hydrophobic interactions through the inclusion of the aromatic ring inside the cyclodextrin cavity.

## Figures and Tables

**Table 1:** Experimental run settings and results of the screening design.

Run Order	Chiral Mobile Phase Additive CMPA Type	CMPA Conc. (mM)	pH	Buffer Conc. (mM)	Flow Rate (mL/min)	%B Change	tR_Last_ (min)	α
1	SBE-β-CD	0.6	6	50	0.05	0.04	152.41	1.00
2	HP-β-CD	10	6	50	0.05	0.04	35.17	1.16
3	HP β-CD	0.6	2.5	5	0.05	0.02	69.82	1.27
4	HP-β-CD	10	6	5	0.3	0.02	6.20	1.16
5	SBE-β-CD	0.6	6	5	0.05	0.02	>240	1.00
6	SBE-β-CD	10	6	5	0.3	0.04	70.65	1.14
7	SBE-β-CD	0.6	2.5	5	0.3	0.04	6.68	1.20
8	HP-β-CD	10	2.5	5	0.05	0.04	39.70	1.18
9	HP-β-CD	0.6	6	50	0.3	0.02	6.97	1.19
10	SBE-β-CD	10	2.5	50	0.05	0.02	241.06	1.11
11	SBE-β-CD	10	2.5	50	0.3	0.02	100.35	1.14
12	HP-β-CD	0.6	2.5	50	0.3	0.04	66.12	1.00

**Table 2:** Experimental runs and results of the central composite design (CCD).

Run Order	CMPA Conc. (mM)	Buffer Conc. (mM)	CMPA Type	tR_Last_ (min)	α
1	5.40	52.25	HP-β-CD	11.11	1.32
2	10.46	27.50	HP-β-CD	34.50	1.01
3	5.40	27.50	SBE-β-CD	69.20	1.10
4	5.40	27.50	HP-β-CD	9.62	1.23
5	10.00	50.00	HP-β-CD	7.89	1.25
6	0.80	50.00	SBE-β-CD	18.61	1.00
7	5.40	27.50	HP-β-CD	7.98	1.26
8	5.40	2.75	HP-β-CD	8.58	1.35
9	0.34	27.50	SBE-β-CD	89.16	1.00
10	5.40	27.50	SBE-β-CD	75.71	1.23
11	5.40	27.50	HP-β-CD	9.69	1.22
12	10.00	50.00	SBE-β-CD	9.74	1.00
13	0.80	5.00	HP-β-CD	11.91	1.19
14	0.34	27.50	HP-β-CD	13.45	1.16
15	0.80	50.00	HP-β-CD	10.09	1.24
16	5.40	27.50	HP-β-CD	7.63	1.24
17	10.00	5.00	HP-β-CD	6.43	1.17
18	5.40	27.50	SBE-β-CD	12.19	1.12
19	5.40	2.75	SBE-β-CD	42.30	1.23
20	10.46	27.50	SBE-β-CD	24.01	1.22
21	10.00	5.00	SBE-β-CD	8.14	1.00
22	5.40	27.50	SBE-β-CD	65.12	1.22
23	5.40	27.50	HP-β-CD	9.38	1.25
24	0.80	5.00	SBE-β-CD	6.50	1.07
25	5.40	27.50	SBE-β-CD	45.58	1.20
26	5.40	52.25	SBE-β-CD	56.04	1.19

**Table 3:** Factors and levels included in the Plackett-Burmann screening design.

Factor Level	CMPA Type	CMPA Conc. (mM)	pH	Buffer Conc. (mM)	Flow Rate (mL/min)	%B Change/min
Low (−1)	HP-β-CD	0.6	2.5	5	0.05	0.02
High (+1)	SBE-β-CD	10	6	50	0.3	0.04

## Supplementary Materials

The following are available online at https://www.mdpi.com/1424-8247/13/11/364/s1, Figure S1: Graphical output of the screening design of TER chiral separation. (A) Main effect plots for retention time (tR_Last_), (B) Main effect plots for selectivity (α). Steepness of the slope in CMPA type and concentration, and buffer concentration indicates the high influence of these studied factors on the responses. Figure S2: Pareto charts expressing the effect of each studied factor on responses: (A) Most influential factors affecting tR_Last_, (B) Most influential factors affecting α.
