# Supplementary material for: Quality-by-Design Is a Tool for Quality Assurance in the Assessment of Enantioseparation of a Model Active Pharmaceutical Ingredient
Source: Pharmaceuticals (Basel). 2020 Nov 4;13(11):364. doi: 10.3390/ph13110364 (PMC7694297; doi:10.3390/ph13110364)
Supplement: Supplementary file 1 [file pharmaceuticals-13-00364-s001.pdf]

pharmaceuticals

Supplementary Materials

Quality-by-Design Is a Tool for Quality Assurance in the Assessment of Enantioseparation of a Model Active Pharmaceutical Ingredient

Dina Aboushady [1], Maria Kristina Parr [2,*] and Rasha S. Hanafi [1]

[1] Department of Pharmaceutical Chemistry, Faculty of Pharmacy and Biotechnology, German University in Cairo, Cairo 11835, Egypt; dina.abou-shady@guc.edu.eg (D.A.); rasha.hanafi@guc.edu.eg (R.S.H.)
[2] Institute of Pharmacy, Freie Universität Berlin, Königin-Luise-Str. 2 + 4, 14195 Berlin, Germany
* Correspondence: maria.parr@fu-berlin.de; Tel: +49-(0)-30-838-57-686

Received: 2 October 2020; Accepted: 31 October 2020; Published: 4 November 2020

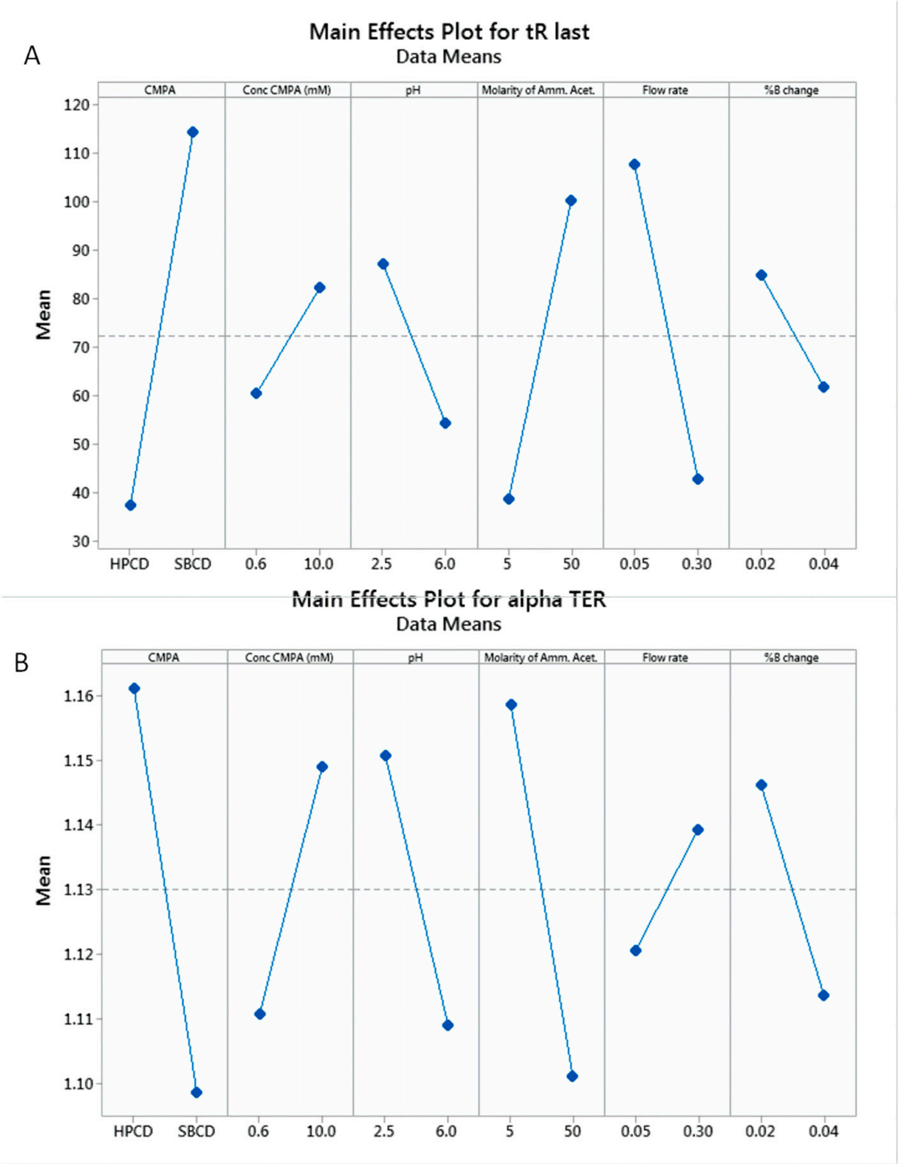

Figure S1. Graphical output of the screening design of TER chiral separation. (**A**) Main Effect Plots for retention time (tR_{Last}), (**B**) Main Effect Plots for selectivity (α). Steepness of the slope in CMPA type and concentration and buffer concentration indicates the high influence of these studied factors on the responses.

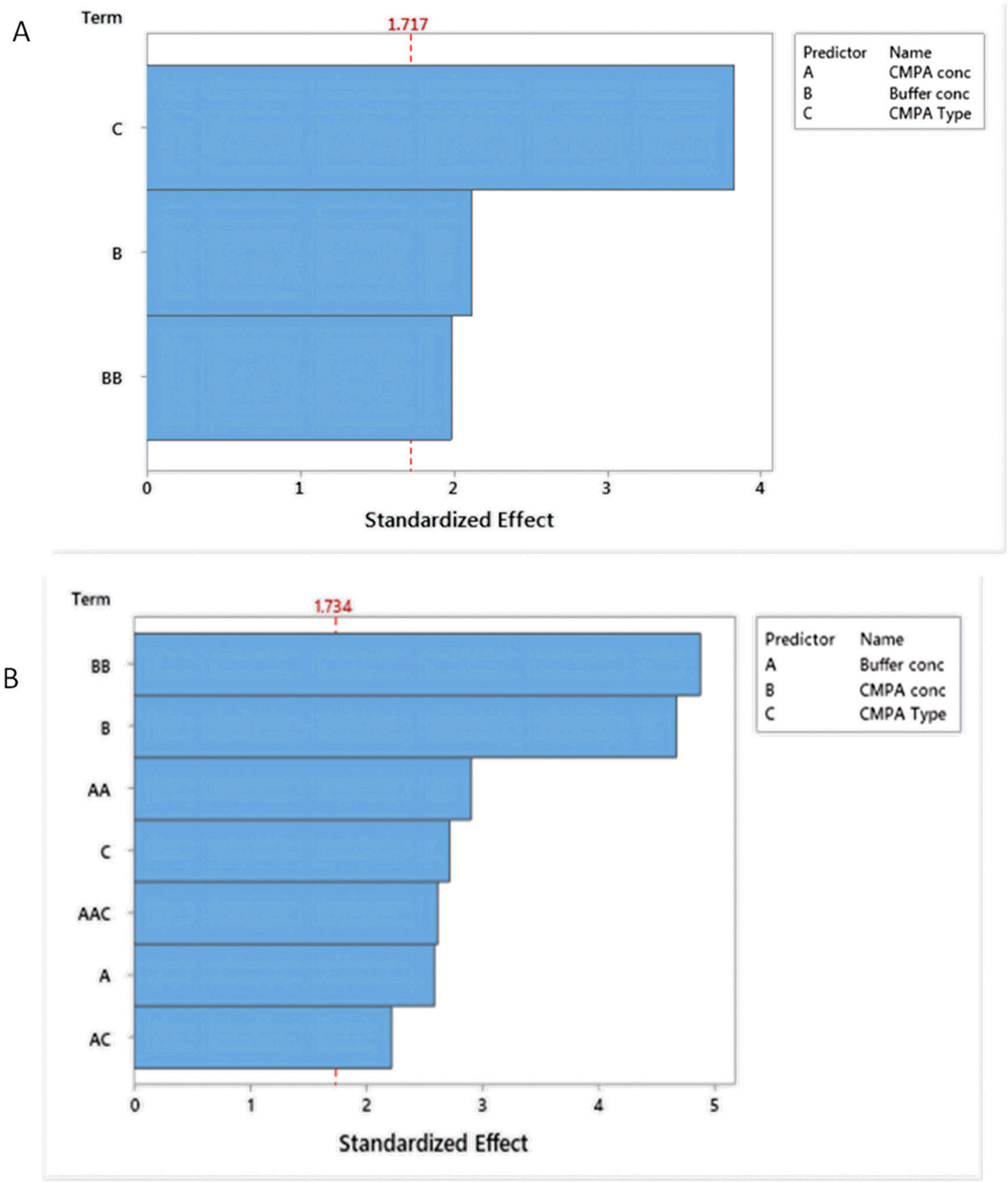

Figure S2. Pareto charts expressing the effect of each studied factor on responses: (**A**) Most influential factors affecting tR_{Last} (**B**) Most influential factors affecting α.
